# The association between maternal urinary phthalate concentrations and blood pressure in pregnancy: The HOME Study

**DOI:** 10.1186/s12940-015-0062-3

**Published:** 2015-09-17

**Authors:** Erika F. Werner, Joseph M. Braun, Kimberly Yolton, Jane C. Khoury, Bruce P. Lanphear

**Affiliations:** Women & Infants’ Hospital, Warren Alpert Medical School at Brown University, 101 Dudley St., Providence, RI 02906 USA; Department of Epidemiology, Brown University School of Public Health, Providence, RI USA; Department of Pediatrics, Cincinnati Children’s Hospital Medical Center, Cincinnati, OH USA; Department of Health Sciences, Simon Fraser University, Vancouver, BC Canada

**Keywords:** Blood pressure, Hypertension, Preeclampsia, Endocrine disruptor, Phthalate

## Abstract

**Background:**

Exposure to phthalates, a class of endocrine disrupting chemicals, is ubiquitous. We examined the association of urinary phthalate metabolite concentrations during pregnancy with maternal blood pressure and risk of pregnancy-induced hypertensive diseases.

**Methods:**

We used data from the Health Outcomes and Measures of the Environment Study, a prospective birth cohort of low-risk pregnant women recruited between March 2003 and January 2006. We analyzed maternal urine samples collected at 16 and 26 weeks gestation for 9 phthalate monoester metabolites reflecting exposure to 6 phthalate diesters. Outcomes included maternal blood pressure at <20 and ≥20 weeks gestation and pregnancy induced hypertensive diseases (gestational hypertension, preeclampsia, eclampsia, and HELLP syndrome).

**Results:**

Data were available for 369 women who gave birth to singleton, live-born infants without congenital anomalies. Of the phthalate metabolites evaluated, only mono-benzyl phthalate (MBzP) concentrations were significantly associated with maternal diastolic blood pressure at <20 weeks gestation. Women in the third MBzP tercile at 16 weeks gestation had diastolic blood pressure 2.2 (95 % CI: 0.5–3.9) mm Hg higher at <20 weeks gestation and 2.8 (95 % CI: 0.9–4.7) mm Hg higher at ≥20 weeks gestation compared to women in the first tercile. Compared to women in the first tercile, women in the top MBzP tercile at 16 weeks had an increased risk of developing pregnancy-induced hypertensive diseases (RR = 2.92, 95 % CI 1.15–7.41, p-value for trend = 0.01). MBzP concentrations at 26 weeks gestation were not as strongly associated with blood pressure at ≥20 weeks gestation or risk of pregnancy-induced hypertensive diseases.

**Conclusion:**

This study suggests that maternal urinary MBzP concentrations may be associated with increased diastolic blood pressure and risk of pregnancy-induced hypertensive diseases.

## Background

Recent studies have suggested that exposure to phthalic acid diesters (i.e., phthalates) may be associated with elevated blood pressure and increased risk of cardiovascular disease. A cross-sectional analysis of the National Health and Nutrition Examination Survey suggested that increased urinary phthalate concentrations are associated with higher blood pressure in children [1]. Additionally, a cross-sectional study of older adults in Sweden found that increased serum concentrations of monobenzyl phthalate (MBzP) were associated with several cardiovascular risk factors [2]. If phthalate exposure impacts blood pressure or cardiovascular risk, it may also impact gestational blood pressure and preeclampsia risk.

Women of reproductive age in the United States have almost ubiquitous exposure to phthalates [3, 4]. Phthalate diesters can be found in polyvinyl chloride plastics, personal care products (e.g., shampoo, soap, perfume), flooring, adhesives, and food processing equipment. Exposure to phthalates can occur through dermal absorption, ingestion, or inhalation. Phthalates are metabolized into their respective monoester metabolites after exposure and then conjugated before being excreted in the urine; thus, urinary phthalate monoester metabolites can be used as sensitive and specific biomarkers of exposure [5].

While the exact mechanism by which phthalate exposure may influence cardiovascular health is unknown, phthalates are hypothesized to be endocrine disruptors that affect the action and metabolism of androgens, cortisol, and thyroid hormones [6–8]. Phthalates may also cause a pro-inflammatory response and increased oxidative stress [9]. This latter mechanism is implicated as a possible etiology for preeclampsia. Thus, we sought to investigate the relationship of maternal urinary phthalate concentrations during gestation with maternal blood pressure and pregnancy-induced hypertensive diseases, such as preeclampsia. We hypothesized that increased maternal urinary phthalate levels would be associated with increased maternal blood pressure and risk of pregnancy-induced hypertensive diseases (gestational hypertension, preeclampsia, eclampsia, and HELLP syndrome).

## Methods

This study is an analysis of data from The Health Outcomes and Measures of the Environment (HOME) Study, a prospective pregnancy and birth cohort study designed to examine the associations of environmental chemicals with maternal and child health outcomes. The HOME Study recruited pregnant women from 2003 to 2006 who were ≥ 18 years of age, 16 ± 3 weeks of gestation, and living in the Cincinnati, OH area in homes built before 1978 [10]. Women were recruited from nine prenatal clinics associated with three hospitals. Women with HIV, or those taking medications for seizures or thyroid disorders were excluded as one of the primary outcomes of the original HOME Study was neurodevelopment. In total, 468 pregnant women enrolled in the HOME Study. The study was approved by the institutional review boards of Cincinnati Children’s Hospital Medical Center, cooperating delivery hospitals, and the Centers for Disease Control and Prevention (CDC).

This analysis included women who were still participating in the study at the time of delivery and who delivered a live singleton (*n* = 389). We excluded two women whose offspring had congenital or chromosomal abnormalities as some fetal conditions (i.e. hydrops) can predispose women to hypertension in pregnancy. Finally, we excluded 18 women who were missing exposure (*n* = 5), outcome (*n* = 4), or covariate (*n* = 9) data, leaving 369 pregnancies for our primary analyses.

The primary outcome we evaluated was the first two diastolic and systolic blood pressure measurements in the woman’s medical chart in early-mid pregnancy (<20 weeks). We selected this as the primary outcome since maternal blood pressure in the first and early second trimester affects placentation and is known to predict preeclampsia risk [11, 12]. Additional outcomes included the two highest blood pressures after 20 weeks gestation and physician diagnosed pregnancy-induced hypertensive diseases, including gestational hypertension, preeclampsia, HELLP syndrome, and eclampsia. As part of the original HOME Study data collection, participants’ medical charts were reviewed after delivery, and all relevant pregnancy complications were abstracted. Trained research assistants abstracted diastolic and systolic blood pressures and medical conditions from medical records. The original study protocol involved abstracting the first two blood pressures available in the medical record (<20 weeks) and the two highest blood pressures after 20 weeks. Additional blood pressure data at <20 or after 20 weeks was not available for this analysis.

Study participants were defined as having gestational hypertension, preeclampsia, eclampsia or HELLP syndrome if their provider made the clinical diagnosis and documented it in their medical record. Additionally, we considered a woman to have gestational hypertension if either of her two blood pressure measurements at ≥ 20 weeks met at least one of the following conditions: elevated systolic blood pressure (>140 mm Hg) at both measurements, elevated diastolic blood pressure (>90 mm Hg) at both measurements, or elevated systolic blood pressure at one measurement and elevated diastolic blood pressure at the other measurement or vice versa. A woman was considered to have a pregnancy-induced hypertensive disease if she had one or more of the following diagnoses: gestational hypertension, preeclampsia, eclampsia or HELLP syndrome. Women with two blood pressures ≥140/90 prior to 20 weeks (chronic hypertension) could still be considered to have a pregnancy-induced hypertensive disease if they developed preeclampsia, eclampsia or HELLP (i.e. superimposed preeclampsia).

We collected covariate information during home visits ~ 20 weeks gestation and at the prenatal visit at the time women completed their glucose tolerance test (~26 weeks gestation). Study staff surveyed participants’ about their socio-demographic status, including maternal age, race, income, marital status and insurance status using standardized interviews. Additionally, we obtained data on parity and body mass index (BMI) at 16 weeks from the medical record. We measured serum cotinine, a biomarker of tobacco smoke exposure, and blood lead in samples collected at approximately 16 and 26 weeks gestation. We also asked women if they had ever taken medication for high blood pressure.

Maternal urinary phthalate metabolite concentrations were obtained from spot samples at an average of 16 (range: 10–23) and 26 (range: 19–35) weeks gestation. Less than 1 % (*n* = 3) of the 16 week samples were collected after 20 weeks of gestation. We collected urine into polypropylene specimen cups that had been lot tested for phthalate contaminants, refrigerated until processing, and stored at or below −20 °C until chemical analysis. Urinary phthalate metabolites were measured using previously described analytic methods [13]. We measured nine phthalate monoester metabolites that reflected exposure to six parent diesters [5]. For phthalate metabolite levels reported as below level of detection (LOD), a value of LOD/√2 was used for analytic purposes. We summed the molar concentrations of DEHP (∑DEHP) and di-butyl phthalate (**∑**DBP) monoester metabolites. Thus, we examined five different phthalates in the analysis: monoethyl phthalate (MEP), mono-3-carboxylpropyl phthalate (MCPP), monobenzyl phthalate (MBzP), ∑DEHP, and **∑**DBP. Phthalate metabolite concentrations were creatinine-normalized to account for maternal hydration by dividing urinary phthalate metabolite concentrations by urinary creatinine concentrations and multiplying this by 100.

### Statistical analysis

We adjusted for maternal race, age at delivery, household income, education, marital status, serum cotinine concentrations, parity, BMI at 16 weeks gestation, and self-reported use of medications for high blood pressure in all of our statistical models. We examined serum lead concentrations as a confounder, but it did not substantially change our estimates, so we did not include it in our final models. Our models examining continuous blood pressure measurements during pregnancy also include the week of gestation to account for changes in blood pressure across the course of pregnancy. We used linear mixed models with an unstructured covariance matrix and random intercept to estimate the association between maternal urinary phthalate metabolite concentrations at 16 weeks and the two repeated maternal blood pressure measures collected at <20 weeks gestation. We examined the association between phthalate metabolite concentrations at 16 and 26 weeks, as well as their average, and the two highest blood pressures at ≥20 weeks gestation using these same models. Finally, we examined phthalate metabolite concentrations at 16 and 26 weeks, as well as their average, and the risk of pregnancy-induced hypertensive diseases using Poisson regression. We examined both continuous log_10_-transformed creatinine-normalized urinary phthalate metabolite concentrations, as well as terciles. We calculated robust standard errors in all of our analyses.

## Results

On average, study participants were well-educated (50 % with at least a bachelor’s degree), married (66 %), and Caucasian (62 %) (Table 1). The average age of participants at delivery was 29.5 years (standard deviation = 5.8 years). All women had detectable levels of at least one phthalate metabolite in their urine; the frequency of detection at 16 or 26 weeks ranged from 75 % (MEHP, level of detection (LOD) 1.2 ng/mL) to 100 % (MEP, LOD 0.5 ng/mL). Most urine phthalate metabolite concentrations at 16 weeks gestation (i.e., baseline) were similar among women who remained enrolled in the study and those who dropped out prior to delivery; ΣDBP (31 vs. 27 μg/g Cr), ΣDEHP (99 vs. 89 μg/g Cr), MCPP (2.5 vs. 2.7 μg/g Cr), and MBzP (9.2 vs. 11.2 μg/g Cr) . MEP (139 vs. 187 μg/g Cr) concentrations were slightly higher among women who dropped out prior to delivery.

**Table 1:** Number of women with pregnancy-induced hypertensive disorders (cases include gestational hypertension, preeclampsia, HELLP syndrome or eclampsia), median (25^th^, 75^th^) urinary phthalate metabolite concentrations (μg/g creatinine), and mean (SD) systolic and diastolic blood pressures (mm Hg) at <20 weeks pregnancy according to covariates among HOME Study women*

Demographic or Perinatal Characteristic	Cases	N (%)^a^	MEP	MBzP	MCPP	∑DBP	∑DEHP	SBP	DBP
Overall	34	369 (9)	132 (70, 285)	9 (5.4, 16)	2.2 (1.6, 3.3)	31 (22, 45)	83 (53, 159)	110 (13)	66 (10)
Race									
White	14	230 (6)	127 (64, 247)	8.2 (5, 14)	2.3 (1.7, 3.4)	31 (22, 44)	98 (59, 191)	111 (12)	68 (10)
Non-White	20	139 (14)	139 (85, 344)	11 (6.4, 21)	1.9 (1.2, 2.9)	32 (21, 51)	74 (48, 125)	109 (13)	63 (10)
Maternal Age at Delivery (years)									
<25	12	88 (14)	133 (79, 292)	13 (7.5, 22)	2 (1.3, 3.2)	34 (21, 56)	66 (47, 124)	109 (12)	63 (10)
25- < 35	18	220 (8)	132 (65, 284)	8.3 (4.9, 15)	2.3 (1.7, 3.3)	30 (20, 43)	94 (58, 180)	111 (13)	67 (10)
35+	4	61 (7)	139 (64, 260)	8 (5.3, 12)	2.1 (1.6, 2.9)	34 (26, 45)	100 (58, 177)	109 (13)	70 (9)
Maternal Education									
Completed College	14	186 (8)	126 (55, 254)	7 (4.8, 12)	2.3 (1.7, 3.3)	30 (21, 42)	98 (58, 197)	111 (13)	68 (9)
Some College	8	94 (9)	135 (71, 303)	11 (5.9, 15)	2.3 (1.6, 3.3)	33 (22, 47)	80 (58, 148)	109 (13)	65 (10)
High School or Less	12	89 (13)	148 (92, 344)	14 (7.9, 24)	2 (1.2, 2.8)	34 (22, 58)	69 (48, 125)	111 (12)	63 (11)
Marital Status									
Married	18	243 (7)	128 (63, 264)	8 (5, 13)	2.3 (1.7, 3.3)	31 (22, 43)	96 (59, 193)	110 (13)	68 (10)
Unmarried-Living Together	8	53 (15)	168 (103, 458)	11 (6.6, 22)	1.8 (1.1, 3.2)	32 (20, 57)	74 (48, 142)	112 (12)	65 (9)
Unmarried-Living Alone	8	73 (11)	131 (75, 285)	13 (7.4, 22)	1.9 (1.3, 2.8)	34 (21, 54)	66 (49, 125)	108 (11)	62 (10)
Household Income									
$80,000+	4	102 (4)	119 (64, 247)	7.2 (4.9, 12)	2.3 (1.7, 3.3)	30 (23, 44)	96 (61, 181)	109 (13)	68 (9)
$40-80,000	14	124 (11)	121 (61, 268)	7.9 (4.6, 12)	2.3 (1.7, 3.2)	31 (20, 40)	97 (58, 205)	111 (12)	68 (9)
$20-40,000	4	63 (6)	147 (70, 336)	9.9 (5.6, 15)	1.7 (1.2, 3.4)	30 (19, 51)	84 (46, 156)	110 (14)	65 (11)
<$20,000	12	80 (15)	148 (88, 347)	15 (9.9, 29)	2.2 (1.4, 3)	36 (23, 58)	73 (48, 115)	109 (11)	63 (10)
Maternal Tobacco Smoke Exposure: Serum Cotinine (ng/mL)									
None: < <0.015	9	137 (7)	111 (56, 264)	6.9 (4.4, 12)	2.2 (1.7, 3.2)	30 (19, 42)	86 (55, 167)	111 (13)	69 (10)
Secondhand: 0.015-3	21	193 (11)	134 (74, 274)	11 (6.1, 17)	2.2 (1.5, 3.3)	32 (22, 47)	82 (55, 162)	110 (12)	65 (10)
Active: >3	4	39 (10)	173 (100, 472)	13 (7.9, 21)	2.3 (1.5, 3.3)	36 (23, 54)	74 (49, 139)	109 (12)	61 (10)
Maternal BMI at 16 weeks (kg/m^2^)									
<25	6	157 (4)	131 (74, 286)	8.8 (5.2, 15)	2.1 (1.6, 3.3)	31 (23, 43)	83 (54, 153)	107 (13)	65 (11)
25- < 30	12	120 (10)	133 (71, 271)	8.2 (5.1, 13)	2.2 (1.5, 3.3)	29 (20, 42)	87 (48, 175)	111 (12)	67 (9)
30+	16	92 (17)	139 (66, 328)	11 (6.3, 21)	2.3 (1.6, 3.1)	36 (24, 57)	81 (58, 150)	113 (12)	68 (10)
Parity									
0	21	166 (13)	140 (83, 319)	7.9 (4.9, 15)	2.3 (1.6, 3.4)	32 (21, 46)	97 (53, 167)	110 (13)	66 (10)
1	4	115 (3)	122 (65, 218)	11 (6.4, 15)	2.3 (1.6, 3.3)	32 (22, 47)	87 (55, 193)	111 (11)	67 (11)
2+	9	88 (10)	130 (63, 305)	10 (5.3, 17)	1.9 (1.3, 2.8)	31 (21, 41)	74 (49, 129)	110 (13)	66 (10)
Medication for High Blood Pressure									
No	28	349 (8)	131 (70, 283)	9.3 (5.4, 16)	2.2 (1.6, 3.3)	32 (22, 45)	86 (52, 162)	109 (12)	66 (10)
Yes	6	20 (30)	192 (89, 364)	8.2 (4.4, 13)	1.9 (1.4, 2.4)	30 (22, 50)	70 (56, 100)	120 (13)	76 (13)

a-Row percent

*∑DBP is the weighted molar sum of MnBP (molecular weight = 222 g/mol) and MiBP (molecular weight = 222 g/mol) concentrations expressed in concentrations of μg/g creatinine. We multiplied the molar sum (μmol/g creatinine) of MnBP and MiBP by the molecular weight of MnBP (222 g/mol). ∑DEHP is the weighted molar sum of MEHP (molecular weight = 272), MEHHP (molecular weight = 294), MEOHP (molecular weight = 292), and MECPP (molecular weight = 308) concentrations expressed in concentrations of μg/g creatinine. We multiplied the molar sum (μmol/g creatinine) of MEHP, MEHHP, MEOHP, and MECPP by the molecular weight of MECPP (308 g/mol)

The phthalate concentrations are the average of the two log_10_-transformed concentrations from 16 and 26 weeks gestation

The average systolic and diastolic blood pressures were 110 (SD: 13) and 66 (SD: 10) mm Hg, respectively, and were first measured at an average of 11 weeks gestation (range 4–20). Thirteen women had blood pressures consistent with chronic hypertension (2 blood pressures ≥ 140/90 before 20 weeks gestation). A total of 34 (9.2 %) women developed at least one the following: gestational hypertension (*n* = 13, 4 %), preeclampsia (*n* = 21, 6 %), or HELLP syndrome (*n* = 2, 1 %). No women were diagnosed with eclampsia. Both of the women with HELLP were also diagnosed with preeclampsia. The women who developed pregnancy-induced hypertensive disorders were most often non-white, overweight or obese, nulliparous, or reported previously taking high blood pressure medications (Table 1).

**Fig. 1:**
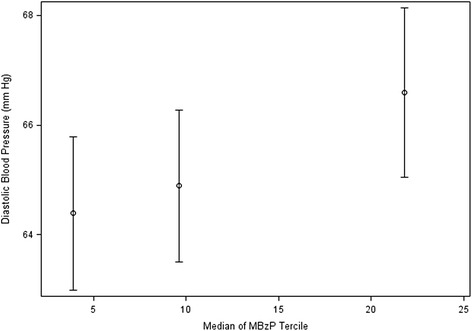
Adjusted diastolic blood pressure at <20 weeks gestation according to urinary MBzP tercile at 16 weeks of pregnancy (*n* = 369, 719 repeated measurements). *- Adjusted for maternal race, age at delivery, household income, education, marital status, serum cotinine concentrations, parity, BMI at 16 weeks gestation, previous use of medication for high blood pressure, and week of pregnancy that the blood pressure measurement was taken

**Fig. 2:**
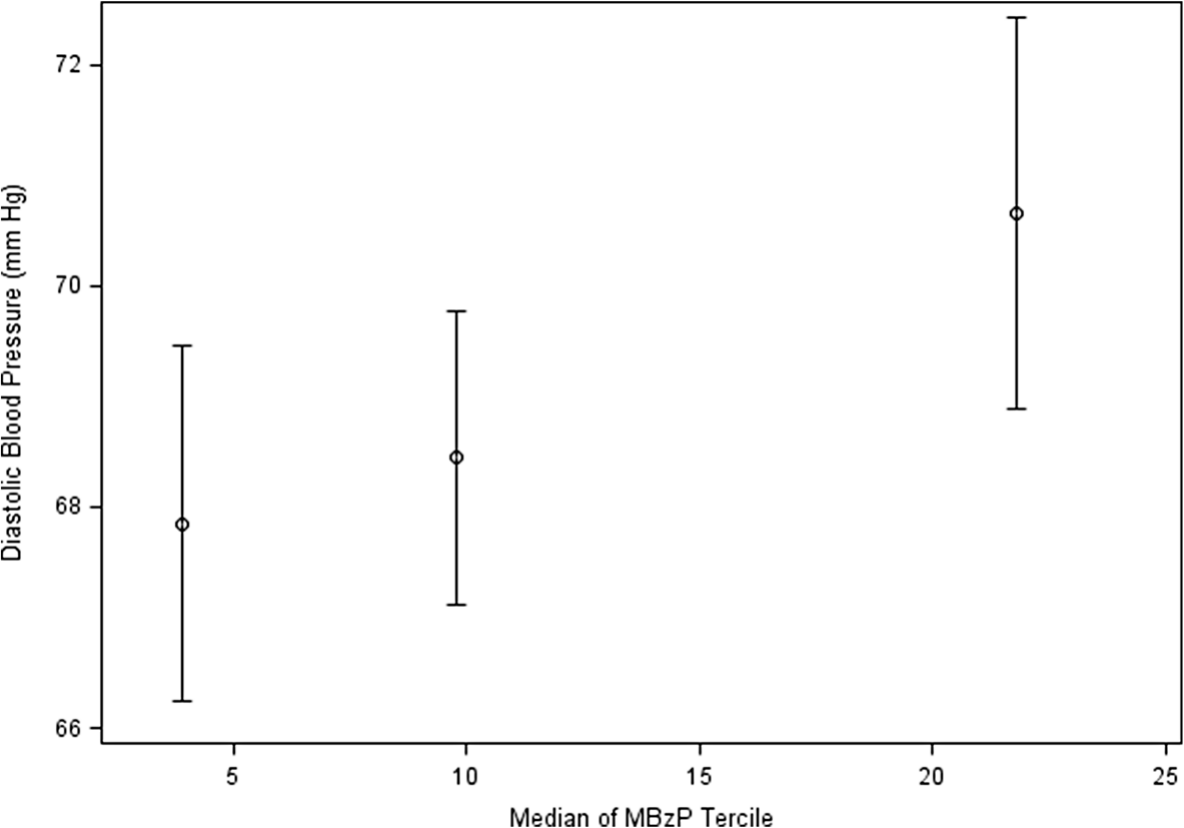
Highest diastolic blood pressure >20 weeks gestation according to urinary MBzP tercile at 16 weeks of pregnancy (*n* = 371, 736 repeated measurements). *- Adjusted for maternal race, age at delivery, household income, education, marital status, serum cotinine concentrations, parity, BMI at 16 weeks gestation, previous use of medication for high blood pressure, and week of pregnancy that the blood pressure measurement was taken

Increasing urinary MBzP concentrations at 16 weeks gestation were significantly associated with increased diastolic blood pressure at <20 weeks gestation (Table 2). Women in the third MBzP tercile at 16 weeks gestation had diastolic blood pressure 2.2 (95 % CI: 0.5-3.9) mm Hg higher at <20 weeks gestation and 2.8 (95 % CI: 0.9-4.7) mm Hg higher at ≥20 weeks gestation compared to women in the first tercile (Figs. 1 and 2, p-value for trend 0.01 and <0.01 respectively). No other phthalate metabolites were significantly associated with blood pressure measures before or after 20 weeks gestation.

**Table 2:** Adjusted difference in blood pressure (mmHg) at < 20 weeks of pregnancy, difference in highest blood pressure (mm Hg) at ≥ 20 weeks of pregnancy, and relative risk of pregnancy induced hypertensive disorders (gestational hypertension, preeclampsia, HELLP syndrome or eclampsia) per 10-fold increase in creatinine-normalized urinary phthalate concentrations during pregnancy*

Phthalate Metabolite and Timing of Urine Measure	Difference in Diastolic BP at <20 Weeks (95 % CI) ^a^	*P*-value	Difference in Systolic BP at <20 Weeks (95 % CI) ^a^	*P*-value	Difference in Diastolic BP at ≥20 Weeks (95 % CI)^b^	*P*-value	Difference in Systolic BP at ≥20 Weeks (95 % CI)^b^	*P*-value	RR of Pregnancy Induced Hypertensive Disorder (95 % CI)^c^	*P*-value
MEP-16 Weeks	−0.1 (−1.3, 1.2)	0.91	0.8 (−1.1, 2.7)	0.40	1.1 (−0.3, 2.5)	0.13	1.0 (−1.2, 3.2)	0.36	1.16 (0.66, 2.05)	0.60
MBzP-16 Weeks	2.3 (0.9, 3.7)	<0.01	1.9 (−0.3, 4.1)	0.08	1.4 (−0.5, 3.3)	0.15	1.3 (−1.4, 4.1)	0.35	1.74 (0.78, 3.89)	0.18
MCPP-16 Weeks	1.1 (−0.9, 3.2)	0.29	0.6 (−2.5, 3.6)	0.72	0.7 (−1.7, 3.0)	0.59	2.4 (−0.9, 5.7)	0.16	0.86 (0.33, 2.24)	0.76
∑DBP-16 Weeks	1.6 (−0.3, 3.5)	0.09	0.5 (−2.5, 3.6)	0.73	1.1 (−1.2, 3.5)	0.34	0.1 (−3.7, 3.8)	0.98	1.42 (0.53, 3.79)	0.48
∑DEHP-16 Weeks	−0.4 (−1.6, 0.8)	0.49	−0.2 (−2.0, 1.6)	0.85	−0.8 (−2.2, 0.5)	0.24	−1.0 (−3.0, 1.0)	0.35	0.85 (0.46, 1.58)	0.61
MEP-26 Weeks					1.0 (−0.4, 2.3)	0.16	0.2 (−1.7, 2.1)	0.83	1.17 (0.67, 2.05)	0.57
MBzP-26 Weeks					1.0 (−0.7, 2.8)	0.25	0.6 (−2.3, 3.4)	0.70	1.59 (0.82, 3.06)	0.17
MCPP-26 Weeks					0.1 (−2.5, 2.6)	0.97	−0.9 (−4.7, 3.0)	0.66	2.73 (1.07, 6.93)	0.04
∑DBP-26 Weeks					2.8 (0.4, 5.3)	0.02	2.7 (−0.9, 6.3)	0.14	2.11 (0.91, 4.90)	0.08
∑DEHP-26 Weeks					0.3 (−1.3, 1.9)	0.73	−0.8 (−3.1, 1.6)	0.52	1.42 (0.64, 3.13)	0.39
MEP-Average					1.4 (−0.2, 3.0)	0.09	0.8 (−1.6, 3.1)	0.53	1.14 (0.59, 2.18)	0.70
MBzP-Average					1.5 (−0.6, 3.6)	0.16	1.1 (−2.0, 4.3)	0.49	1.98 (0.96, 4.11)	0.07
MCPP-Average					0.6 (−2.4, 3.7)	0.69	1.1 (−3.3, 5.5)	0.62	1.67 (0.58, 4.82)	0.34
∑DBP-Average					2.8 (−0.1, 5.8)	0.06	1.7 (−2.7, 6.1)	0.45	2.49 (0.81, 7.65)	0.11
∑DEHP-Average					−0.6 (−2.4, 1.3)	0.55	−1.6 (−4.3, 1.2)	0.27	1.09 (0.45, 2.66)	0.84

*-Adjusted for maternal race, maternal age at delivery, household income, education, marital status, serum cotinine concentration, weeks of gestation at blood pressure measurement, parity, BMI at 16 weeks gestation, and previous use of blood pressure medications. The blood pressure models were additionally adjusted for the week of pregnancy that the blood pressure measurement was taken

a-*n* = 369 with 719 repeated observations

b-*n* = 371 with 736 repeated observations for 16 weeks, *n* = 360 with 714 repeated observations for 26 weeks, and *n* = 374 with 742 repeated observations for average

c-*n* = 366 for 16 week, 355 for 26 week, and 369 for average

We observed a trend suggesting increased risk of pregnancy induced hypertensive disease with higher 16 week, 26 week, or average urinary MBzP concentrations (p-values for trend = 0.01, 0.12, and 0.04, respectively) . The association was strongest for the 16 week urinary MBzP concentrations. Women in the 3^rd^ tercile of urinary MBzP concentrations were almost three times as likely to have a pregnancy-induced hypertensive disease compared to women in the lowest tercile (RR 2.92; 95 % CI 1.15-7.41) (Table 3).

**Table 3:** Adjusted difference in blood pressure (mmHg) at < 20 weeks of pregnancy, difference in highest blood pressure (mm Hg) at ≥ 20 weeks of pregnancy, and relative risk for pregnancy-induced hypertensive disease (gestational hypertension, preeclampsia, HELLP syndrome or eclampsia) according to terciles of creatinine-normalized urinary MBzP concentrations*

MBzP Tercile and Timing of Urine Sample	Cases/Total	Median (Range) MBzP Concentration (μg/g Cr)	Difference in Diastolic BP at <20 Weeks (95 % CI) ^a^	Difference in Systolic BP at <20 Weeks (95 % CI) ^a^	Difference in Diastolic BP at ≥20 Weeks (95 % CI)^b^	Difference in Systolic BP at ≥20 Weeks (95 % CI)^b^	RR for pregnancy-induced hypertensive disease (95 % CI)^c^
16 Week-1^st^	6/122	3.9 (0.5, 6.5)	Ref	Ref	Ref	Ref	Ref
16 Week-2^nd^	8/122	9.8 (6.5, 13)	0.5 (−1.1, 2.1)	−1.0 (−3.4, 1.5)	0.6 (−1.2, 2.4)	−0.3 (−3.0, 2.4)	1.44 (0.51, 4.02)
16 Week-3^rd^	19/122	22 (13, 331)	2.2 (0.5, 3.9)	2.1 (−0.5, 4.7)	2.8 (0.9, 4.7)	2.3 (−0.3, 4.9)	2.92 (1.15, 7.41)
P-value for trend			<0.01	0.05	<0.01	0.05	0.01
26 Week-1^st^	10/118	3.9 (0.1, 6.0)			Ref	Ref	Ref
26 Week-2^nd^	7/119	9.0 (6.1, 14)			0.9 (−1.0, 2.9)	1.0 (−1.8, 3.8)	0.91 (0.35, 2.42)
26 Week-3^rd^	15/118	23 (14, 178)			1.1 (−0.7, 3.0)	0.6 (−2.2, 3.4)	1.65 (0.76, 3.60)
P-value for trend					0.29	0.80	0.12
Pregnancy Average-1^st^	8/123	4.5 (0.2, 6.4)			Ref	Ref	Ref
Pregnancy Average-2^nd^	9/123	9.0 (6.4, 13)			1.0 (−0.8, 2.8)	1.1 (−1.5, 3.8)	1.30 (0.55, 3.09)
Pregnancy Average-3^rd^	17/123	21 (13, 137)			2.1 (0.3, 3.9)	0.5 (−2.1, 3.1)	2.21 (0.96, 5.09)
P-value for trend					0.03	0.86	0.04

*- Adjusted for maternal race, age at delivery, household income, education, marital status, serum cotinine concentrations, parity, BMI at 16 weeks gestation, and previous use of medication for high blood pressure. The blood pressure models were additionally adjusted for the week of pregnancy that the blood pressure measurement was taken

a-*n* = 369 with 719 repeated observations

b-*n* = 371 with 736 repeated observations for 16 weeks, *n* = 360 with 714 repeated observations for 26 weeks, and *n* = 374 with 742 repeated observations for average

c-*n* = 366 for 16 week, 355 for 26 week, and 369 for average

We performed several sensitivity analyses to examine the robustness of our MBzP associations to various assumptions. First, given the potential for urinary phthalate concentrations to be significantly affected by renal function, we excluded women with low (<20 mg/dL) or high (>300 mg/dL) 16 week urinary creatinine concentrations (*n* = 28). The association between MBzP concentrations and maternal diastolic blood pressure <20 weeks did not change appreciably after this exclusion. Second, we accounted for urine dilution by adjusting for creatinine concentrations in our statistical models of 16 and 26 week urinary phthalate metabolites, rather than creatinine-normalizing urinary phthalate concentrations; again our results were relatively unchanged. Third, we also analyzed the data stratifying for the timing of blood pressure measurements prior to 20 weeks gestation relative to the timing of urine sample collection. We found a consistent association between MBzP concentrations and increased diastolic blood pressure at <20 weeks of pregnancy regardless of whether the blood pressure measurements were obtained before or after the urine sample collection. Finally, we excluded women who reported previously using medication for high blood pressure (*n* = 20) and our results were unchanged.

## Discussion

In this prospective, longitudinal pregnancy and birth cohort, we found that urinary MBzP concentrations in early-mid pregnancy were significantly associated with increased diastolic blood pressure and an increased risk of pregnancy-induced hypertensive diseases. The associations between urinary MBzP concentrations and diastolic blood pressure in this study are modest, but the population impact could be substantial because exposure to the parent compound of this metabolite, butyl benzyl phthalate, is ubiquitous in U.S. women [4].

A study using data from the National Health and Nutrition Examination Survey (NHANES) found that increased urinary concentrations of MEP were associated with increased blood pressure in children age 6 to 19 years (OR per log-unit increase:1.20; 95 % confidence interval 1.01-1.43) [1]. In another study, higher urinary concentrations of the DEHP metabolite, MECPP in early pregnancy (4.7-16.1 weeks) were associated with placental causes of preterm birth (OR 1.46; 95 % CI 1.1-1.95) among US women [14]. In the same study, concentrations of other phthalate metabolites, including MBzP were associated with preterm birth, but when placental-causes of preterm birth were examined separately, the results did not reach statistical significance [14].

In our study, we did not find an association between MEP or DEHP and maternal blood pressure or pregnancy-induced hypertensive disease. Only MBzP showed a significant association with both increased diastolic blood pressure and pregnancy-induced hypertensive diseases. Serum MBzP has previously been associated with cardiovascular disease risk factors in a study of elderly adults from Sweden [2]. Individuals with higher serum concentrations of MBzP were more likely to have plaque echogenicity and intima-media thickening and echogenicity. However, serum phthalate monoester biomarkers could be subject to exogenous diester phthalate contamination, thus, making them inappropriate for epidemiological studies [15]. It is plausible that similar subtle changes in the vasculature of reproductive age women could lead to slightly higher diastolic blood pressures and an increased risk of pregnancy-induced hypertensive disease. Thus future studies using urinary biomarkers, which are less susceptible to exogenous contamination, might benefit from ultrasound assessment of the uterine arteries in addition to biologic specimen and outcome collection.

This study has several limitations. While the HOME Study collected a rich set of phthalate exposure biomarkers from pregnant women, the primary outcome of interest was not blood pressure or hypertensive disorders of pregnancy. Thus, we relied on chart-derived blood pressure measures rather than research quality blood pressure measures. Blood pressures can be falsely elevated if collected immediately after a patient enters the clinic, after smoking tobacco or if the blood pressure cuff is too small. In addition, we did not have detailed information on the quality controls used to ensure accurate blood pressures. However, this bias should be non-differential with respect to phthalate exposure. Another limitation of this analysis is that we do not have pre-pregnancy health status which may have contributed to the risk of increased blood pressures and pregnancy induced hypertensive diseases. We also evaluated five different types of phthalate metabolites and there is a chance of type I error due to multiple comparisons. However, it seems unlikely that our MBzP associations were spurious given that we saw consistent associations between urinary MBzP concentrations and blood pressure at two time points in pregnancy, as well as risk of pregnancy-induced hypertensive diseases. Furthermore, the MBzP concentration in the earlier pregnancy window (16 weeks rather than 26 weeks) was most strongly tied to pregnancy induced hypertensive disorders. This is consistent with the hypothesis that some pregnancy induced hypertensive disorders are the end result of an abnormal spiral artery invasion between mother and fetus that develops prior to 16 weeks gestation [16].

The generalizability of these findings might be limited since our study participants were at lower-risk of pregnancy-induced hypertensive disorders then most obstetric populations. However, urinary phthalate concentrations among women in this cohort were similar to nationally-representative samples of women in the US [17]. Additionally, while we have sensitive and specific biomarkers of phthalate exposure from two time points during pregnancy, urinary phthalate metabolite concentrations exhibit moderate to high variability within individuals due to their relatively short biological half-life (<24 h) and the episodic nature of exposure. To the extent that phthalate exposures were misclassified, our results would be biased toward the null. Finally, while we can speculate on the mechanism by which phthalate concentrations impact blood pressure and pregnancy induced hypertensive diseases, we do not have data on the cytokine markers of inflammation or other relevant biological pathways.

This study has several strengths. First, it is a prospective cohort study that enrolled women in early pregnancy to investigate the impact of environmental chemicals. In addition, we made every effort to minimize exogenous contamination of our samples and quantified urinary levels of phthalate metabolites using state of the art analytic chemistry methods. This is particularly important because the parent phthalate diesters may be found in specimen collection materials and other medical equipment, thus potentially contaminating the samples [18]. By examining the metabolized phthalate monoesters, we minimized the potential for this exogenous contamination. Second, we collected two measures of phthalate metabolites during pregnancy, which allowed us to reduce some of the within-person variation of these exposures and examine distinct windows of vulnerability. Finally, we controlled for numerous confounders including biomarkers of other environmental exposures, such as serum cotinine and serum lead concentrations that are often not available in other studies. However, it is possible that other chemicals in products containing BBzP (the parent diester of MBzP) may be responsible for some or all of the association between urinary MBzP concentrations and blood pressure or blood-pressure related complications.

## Conclusions

In this cohort, we observed that higher urinary MBzP concentrations were associated with both elevated maternal diastolic blood pressure and increased risk of pregnancy-induced hypertensive diseases. While exposure to MBzP’s parent compound, BBzP, is nearly universal among pregnant women, exposure could be prevented. Thus, it is vital that additional studies more thoroughly evaluate how, and when phthalates can affect maternal and fetal health.
